# Mental Health Status and Fear of COVID-19 in Young Adult Male Inmates in Portugal

**DOI:** 10.1192/j.eurpsy.2024.1238

**Published:** 2024-08-27

**Authors:** C. Laranjeira, W. Baccon, R. Mendes

**Affiliations:** ^1^ School of Health Sciences; ^2^ciTechCare, Polytechnic University of Leiria, Leiria, Portugal; ^3^Nursing Department, State University of Maringá, Maringá, Brazil; ^4^Estabelecimento Prisional de Leiria, Leiria, Portugal

## Abstract

**Introduction:**

Incarcerated individuals are subject to a heightened risk of both mental and physical ailments. Hence, it is important to conduct regular assessments of their mental well-being and other potential health hazards.

**Objectives:**

The aim of this study is to examine the subjective experience of fear related to COVID-19 and the psychological consequences of the pandemic among a cohort of young adult male convicts.

**Methods:**

A research design using an institutional-based quantitative cross-sectional approach was used. The data collecting period was from July to September 2022, during which data was gathered at a juvenile correctional facility located in the center area of Portugal. The researchers used questionnaires to gather data pertaining to demographic and health attributes, fear related to COVID-19, as well as measures of depression, anxiety, stress, and resilient coping.

**Results:**

The study included a cohort of 60 incarcerated males who had been imprisoned for a duration exceeding 2 years. The prevalence of stress was found to be the highest among offenders, with around 75% reporting this symptom. Anxiety was the second most often reported symptom, with 38.3% of inmates experiencing it, followed closely by depression, which was reported by 36.7% of the inmate population. The average score on the Fear of COVID-19 Scale was 17.38 ± 4.80, suggesting that participants generally reported mild levels of fear. A total of 38 subjects, accounting for 63.3% of the sample, had low scores in resilience. The participants’ responses indicated that their perceptions of mental health were within a fairly high range, with an average score of 3.62 ± 0.87. Similarly, their perceptions of physical health were also moderately high, with an average score of 3.73 ± 0.95. In terms of global health, participants reported a slightly lower average score of 3.27 ± 0.82 for the preceding month. The Pearson correlation matrix revealed statistically significant associations between fear of COVID-19 and characteristics linked to mental health, with the strength of these associations ranging from moderate to high (p < 0.001). The identification of predictive variables for fear of COVID-19 was accomplished by the use of a multiple linear regression model. Four predictors were identified in the study, namely age, perception of mental health, and overall levels of anxiety and stress. These predictors together account for about 49.7% of the variance in the outcome variable.

**Conclusions:**

The findings of our research indicate a significant prevalence of stress among incarcerated individuals, accompanied by moderate levels of anxiety and depression. Our research has the potential to provide valuable insights for policymakers, mental health professionals, public health specialists, and other relevant stakeholders in the identification and effective management of pandemic-induced anxieties and mental health symptoms.

**Disclosure of Interest:**

None Declared

